# Development and external validation of preoperative risk models for operative morbidities after total gastrectomy using a Japanese web-based nationwide registry

**DOI:** 10.1007/s10120-017-0706-9

**Published:** 2017-03-11

**Authors:** Hirotoshi Kikuchi, Hiroaki Miyata, Hiroyuki Konno, Kinji Kamiya, Ai Tomotaki, Mitsukazu Gotoh, Go Wakabayashi, Masaki Mori

**Affiliations:** 1 The Japanese Society of Gastroenterological Surgery, Working Group Database Committee, Tokyo, Japan; 2 The Japanese Society of Gastroenterological Surgery, Database Committee, Tokyo, Japan; 3 National Clinical Database, Tokyo, Japan; 4 The Japanese Society of Gastroenterological Surgery, Tokyo, Japan; 5 Hamamatsu University School of Medicine, 1-20-1 Higashi-ku, Hamamatsu, 431-3192 Japan

**Keywords:** Total gastrectomy, Risk model, Morbidity, Mortality, National Clinical Database

## Abstract

**Background:**

Total gastrectomy is a relatively difficult and invasive procedure among gastrointestinal surgeries, and major morbidities following total gastrectomy can be serious and fatal. This study aimed to develop and validate preoperative risk models of morbidities associated with total gastrectomy using a Japanese web-based nationwide registry.

**Methods:**

The national clinical database was used to retrieve the records of 39,253 patients who underwent total gastrectomy in 1,841 hospitals between January 1, 2011 and December 31, 2012.

**Results:**

Mean patient age was 69.1 years, and 73.8% of the patients were male. The overall morbidity rate was 21.5%, which included 8.1% with surgical site infection (SSI), 4.5% with anastomotic leak, 5.0% with pancreatic fistula, 3.7% with pneumonia, 1.9% with prolonged ventilation, and 1.2% with renal failure. Sex, splenectomy, and Brinkman index were selected as common risk factors for SSI, anastomotic leak, and pancreatic fistula. Pancreatectomy was the most significant preoperative risk factor in the risk model of SSI and pancreatic fistula. Need of assistance with activities of daily living, chronic obstructive pulmonary disease, previous cerebrovascular disease, American Society of Anesthesiologists score class 3 and over, presence of esophageal cancer, and body mass index more than 25 were selected as common risk factors for pneumonia, prolonged ventilation over 48 h, and renal failure.

**Conclusions:**

We have created the first reported risk models of morbidities associated with total gastrectomy, using a Japanese nationwide database. The risk models developed in this study may be useful to preoperatively predict operative morbidities in patients undergoing total gastrectomy.

**Electronic supplementary material:**

The online version of this article (doi:10.1007/s10120-017-0706-9) contains supplementary material, which is available to authorized users.

## Introduction

Gastric cancer is the fourth most common cancer, the second most common cancer-related cause of death in the world, and one of the major causes of death in South America, Eastern Europe, and East Asia, including Japan [1, 2]. Surgery is often the most effective and the only curative treatment for early and advanced gastric cancer [3]. Total gastrectomy is usually indicated for tumors located in the upper third of the stomach or advanced gastric cancers extending to the cardia.

Total gastrectomy is a relatively difficult and invasive procedure among gastrointestinal surgeries, and major complications following total gastrectomy such as esophagojejunal anastomotic leakage and pancreatic fistula can be serious and fatal [4]. Gastric cancer patients frequently have anemia, malnutrition, or organ dysfunction resulting from tumor extension, which are thought to be preoperative surgical risks [5]. Recently, preoperative chemotherapy has been developed and the number of elderly patients with gastric cancer is increasing [6–9]; therefore, it seems that an increasing number of gastric cancer patients with preoperative surgical risks undergo surgery, including total gastrectomy. In contrast, in randomized control trials (RCTs) evaluating the short-term outcomes or efficacy of gastrectomy, the inclusion criteria generally exclude such elderly and high-risk patients; therefore, the cohorts in RCTs might not be representative of all patients undergoing gastrectomy nationwide [10, 11]. To date, there are few studies that have used a large patient cohort to describe a risk model of mortality or morbidity for total gastrectomy [12–14]. In addition, although the operative outcomes for gastrectomy have been reported from several high-volume centers [15], the nationwide outcomes in Japan were unknown until recently.

The National Clinical Database (NCD), a nationwide, web-based, data entry system that is linked to the surgical board certification system in Japan, commenced patient registration in January 2011. Using the NCD data in 2011, the nationwide outcomes for several operative procedures including total gastrectomy have been reported, and a total gastrectomy risk model has been developed [13]. This surgical risk model likely represents the current status of total gastrectomy in Japan that is not affected by patient selection bias.

Although operative mortality is obviously the most important clinical endpoint, a focus on morbidity, which impacts a patient’s functional status and quality of life and may result in patient death, is also needed to improve surgical outcome. In this study, we created a risk model of operative morbidities for Japanese patients undergoing total gastrectomy using the NCD data.

## Methods

### Data collection

In 2011 and 2012, the NCD registered 949,824 surgical cases that were in the gastroenterological surgery section. For eight procedures including total gastrectomy that were determined to represent the performance of surgery in each specialty, the input of detailed items such as laboratory data and operative morbidities was requested. The Brinkman index was defined as the number of cigarettes smoked per day multiplied by the number of years of smoking. Patients who declined to have their records entered into the NCD were excluded from our analysis. Records from which data were missing on patient age, sex, or status 30 days after surgery were also excluded. A total of 39,253 patients who underwent total gastrectomy at 1,841 institutions between January 1, 2011 and December 31, 2012 were eligible for analysis. In addition, clinical data of 18,744 patients who underwent total gastrectomy in 2013 were utilized to validate the risk models developed using NCD data in 2011 and 2012.

The NCD ensures traceability of its data by maintaining continuity in the staff who approve data, the staff of the departments in charge of annual cases, and the data entry personnel. It also validates data consistency via random inspections by participating institutions. All variables, definitions, and inclusion criteria for the NCD are accessible to participating institutions on its website (http://www.ncd.or.jp/); the database administrators also provide e-learning systems to teach participants how to input data consistently. The administrators answer all inquiries regarding data entry, and “Frequently Asked Questions” are displayed on the website.

### Endpoint

The primary outcome measure of this study was the preoperative risk factors related to morbidities after total gastrectomy: surgical site infection (SSI), anastomotic leak, pancreatic fistula, pneumonia, prolonged ventilation over 48 h, and renal failure, which were relatively frequent or closely associated with mortality.

### Statistical analysis

Risk models for operative morbidities were developed and validated using the total gastrectomy population registered between 2011 and 2012. First, three surgical and three nonsurgical complications were selected for developing the risk models according to the frequency of each morbidity in the entire study population, impact on 30-day and operative mortality with high Pearson’s correlation coefficient, and complications of clinical importance that could affect the quality of life or duration of hospital stay. Data were randomly assigned to two subsets, with 80% allocated for model development and 20% for validation testing. The development dataset comprised 31,415 records, and the validation dataset comprised 7,838 records. The six sets of logistic models, namely, SSI, anastomotic leak, pancreatic fistula, pneumonia, prolonged ventilation over 48 h, and renal failure, were constructed for the development dataset using a stepwise selection of predictors, with the *p* value for inclusion set at 0.05. A goodness-of-fit test was performed to assess how well the model could discriminate between patients with and without each morbidity. Model calibration, the degree to which the observed outcomes were similar to the predicted outcomes, was examined by comparing the observed with the predicted average within each of the five equal-sized subgroups, arranged in increasing order of patient risk. Risk models of operative morbidities developed and validated using NCD data registered between 2011 and 2012 were further validated using NCD data registered in 2013.

## Results

### Study population risk profile

The total gastrectomy patient population represented in the NCD had an average age of 69.1 years; 73.8% of the population was male. Among them, 2.0% required emergency surgery, 4.6% needed assistance with activities of daily living (ADL), and 21.4% had a smoking habit within 1 year. Weight loss of more than 10% was observed in 8.5% of patients. American Society of Anesthesiologists (ASA) scores of class 3 and class 4/5 were seen in 9.6% and 0.7% of patients, respectively. Preoperative comorbidities included diabetes mellitus in 16.0%, preoperative respiratory distress within 30 days in 2.3%, chronic obstructive pulmonary disease (COPD) in 4.2%, ascites in 2.0%, previous cerebrovascular disease (CVD) in 3.9%, disseminated cancer in 3.2%, and systemic sepsis in 0.4% of patients. An abbreviated demographic and risk profile of the study population is shown in Table 1.

**Table 1:** Key descriptive data

Variables	*n* = 39,253
Age, mean ± SD, years Age category, total <59 years (%) 60–64 years (%) 65–69 years (%) 70–74 years (%) 75–79 years (%) ≥80 years (%) 80–84 years (%) 85–89 years (%) ≥90 years (%) Sex, male (%) Body surface area, mean ± SD, m^2^ Body mass index, category, total <25 (%) 25–30 (%) 30–35 (%) ≥35 (%) Emergent surgery (%) Diabetes mellitus (%) Smoking within a year (%) Habitual alcohol consumption (%) Any alcohol consumption (%) Respiratory distress within 30 days (%) Preoperative ADL, any assistance (%) Preoperative ventilation within 48 h (%) COPD (%) Preoperative pneumonia (%) Ascites within 30 days (%) Ascites, uncontrolled (%) Hypertension within 30 days (%) Hypertension without treatment (%) Congestive heart failure within 30 days (%) Myocardial infarction within 6 months (%) Angina pectoris within 30 days (%) Previous PCI (%) Previous cardiac surgery (%) Previous PVD surgery (%) Previous cerebrovascular disease (%) Previous cerebrovascular accident (%) Acute renal failure within 24 h (%) Preoperative dialysis within 14 days (%) Disseminated cancer (%) Chronic steroid use (%) Weight loss more than 10% within 6 months (%) Bleeding disorder (%) Preoperative transfusion within 72 h (%) Preoperative chemotherapy within 30 days (%) Systemic sepsis (%) Epidural anesthesia (%) ASA score, class 3 and over (%) ASA score, class 4 and 5 (%) Non-cancer surgery (%) Esophageal cancer (%) Gastric cancer (%) Colorectal cancer (%) Gallbladder cancer (%) Metastatic or relapsed cancer (%) Cholecystectomy (%) Splenectomy (%) Pancreatectomy (%)	69.1 ± 11.1 39,253 6,295 (16.0) 5,748 (14.6) 6,201 (15.8) 7,624 (19.4) 7,192 (18.3) 6,193 (15.8) 4,515 (11.5) 1,482 (3.8) 196 (0.5) 28,969 (73.8) 1.58 ± 0.18 37,913 31,651 (83.5) 5,627 (14.8) 534 (1.4) 101 (0.3) 779 (2.0) 6,283 (16.0) 8,393 (21.4) 10,046 (25.6) 20,436 (52.1) 919 (2.3) 1,824 (4.6) 43 (0.1) 1,633 (4.2) 180 (0.5) 804 (2.0) 678 (1.7) 13,141 (33.5) 874 (2.2) 289 (0.7) 285 (0.7) 603 (1.5) 1,063 (2.7) 454 (1.2) 235 (0.6) 1,519 (3.9) 782 (2.0) 34 (0.1) 220 (0.6) 1,257 (3.2) 311 (0.8) 3,333 (8.5) 1,350 (3.4) 1,596 (4.1) 1,587 (4.0) 161 (0.4) 27,303 (69.6) 4,045 (10.3) 277 (0.7) 624 (1.6) 214 (0.5) 37,945 (96.7) 725 (1.8) 41 (0.1) 1,019 (2.6) 7,249 (18.5) 3,061 (7.8) 839 (2.1)

*ADL* activities of daily living, *ASA* American Society of Anesthesiologists, *COPD* chronic obstructive pulmonary disease, *PCI* percutaneous coronary intervention, *PVD* peripheral vascular disease, *SD* standard deviation

### Morbidity

The overall morbidity in the total gastrectomy NCD population was 21.5%. Surgical complications included SSI in 8.1%, anastomotic leak in 4.5%, pancreatic fistula (grades A, B, and C) in 5.0%, and wound dehiscence in 0.9%. Nonsurgical complications included pneumonia in 3.7%, unplanned intubation in 1.7%, prolonged ventilation over 48 h in 1.9%, renal failure in 1.2%, central nervous system events in 0.8%, cardiac events in 0.6%, and systemic sepsis in 3.0% (Table 2).

**Table 2:** Prevalence of morbidity associated with total gastrectomy outcomes

Complications	Entire study population (*n* = 39,253)		Total gastrectomy outcomes groups							
			30-day mortality (*n* = 385, 1.0%)				Operative mortality (*n* = 896, 2.3%)			
	*n*	%	*n*	%	*r*	*p* value	*n*	%	*r*	*p* value
Overall complications	8,425	21.5	322	3.8	0.151	<0.001	674	8.0	0.200	<0.001
Reoperation for any reason	1,750	4.5	288	5.0	0.135	<0.001	203	11.6	0.135	<0.001
Surgical complications										
Surgical site infection	3,160	8.1	77	2.4	0.044	<0.001	264	8.4	0.120	<0.001
Superficial incisional	1,178	3.0	28	2.4	0.025	<0.001	113	9.6	0.086	<0.001
Deep incisional	526	1.3	22	4.2	0.038	<0.001	84	16.0	0.107	<0.001
Organ space	2,388	6.1	71	3.0	0.051	<0.001	229	9.6	0.125	<0.001
Anastomotic leak	1,755	4.5	72	4.1	0.069	<0.001	218	12.4	0.147	<0.001
Pancreatic fistula (grade A, B, C)	1,947	5.0	30	1.5	0.013	0.010	94	4.8	0.039	<0.001
Bile leakage	196	0.5	5	2.6	0.011	0.025	22	11.2	0.042	<0.001
Wound dehiscence	345	0.9	19	5.5	0.043	<0.001	62	18.0	0.099	<0.001
Nonsurgical complications										
Pneumonia	1,449	3.7	126	8.7	0.153	<0.001	292	20.2	0.234	<0.001
Unplanned intubation	653	1.7	181	27.7	0.353	<0.001	299	45.8	0.379	<0.001
Pulmonary embolism	55	0.1	6	10.9	0.038	<0.001	10	18.2	0.040	<0.001
Prolonged ventilation over 48 h	735	1.9	167	22.7	0.305	<0.001	302	41.1	0.359	<0.001
Renal failure	473	1.2	115	24.3	0.261	<0.001	215	45.5	0.319	<0.001
Urinary tract infection	267	0.7	10	3.7	0.023	<0.001	43	16.1	0.077	<0.001
Events in the central nervous system	306	0.8	74	24.2	0.209	<0.001	131	42.8	0.241	<0.001
Cardiac events	228	0.6	142	62.3	0.475	<0.001	177	77.6	0.386	<0.001
Transfusion	1,468	3.7	151	10.3	0.186	<0.001	321	21.9	0.258	<0.001
Systemic sepsis	1,172	3.0	145	12.4	0.066	<0.001	320	27.3	0.135	<0.001

*r* Pearson’s correlation coefficient

### Model results

From the surgical complications, SSI, anastomotic leak, and pancreatic fistula were selected as having high frequency (>4%) and high impact on mortality or clinical importance (Table 2). From nonsurgical complications, pneumonia, prolonged ventilation over 48 h, and renal failure were selected as having high frequency (>1%) and a high mortality rate exceeding 20% (number of operative mortality/total number of events) (Table 2). The final logistic models of surgical and nonsurgical complications, with the odds ratios (ORs) and 95% confidence intervals (CIs), are presented in Tables 3 and 4, respectively.

**Table 3:** Risk model of surgical complications

Variables	Status	Surgical site infection		Anastomotic leak		Pancreatic fistula	
		OR	95% CI	OR	95% CI	OR	95% CI
Age category	5 years–up	1.028	1.001–1.056	1.069	1.032–1.107	**–**	**–**
Sex	Male	1.318	1.176–1.478	1.328	1.148–1.535	1.226	1.043–1.441
Preoperative status	Emergent	1.431	1.105–1.854	**–**	**–**	**–**	**–**
Diabetes mellitus	Insulin use	–	**–**	1.307	1.001–1.706	**–**	**–**
Alcohol consumption	Habitual or social	1.155	1.055–1.266	**–**	**–**	1.327	1.183–1.489
Preoperative ADL	Any assistance	1.297	1.088–1.545	1.352	1.089–1.679	–	–
Hypertension	Present within 30 days	1.136	1.040–1.241	1.127	1.004–1.265	–	–
Previous PCI	Performed	–	–	1.363	1.047–1.774		
Previous CVD	Present	1.379	1.152–1.651	–	–		
Steroid	Chronic use	1.527	1.054–2.210	2.089	1.382–3.157		
Weight loss	Over 10%	1.264	1.104–1.446	1.232	1.031–1.471		
Systemic sepsis	Present	2.370	1.527–3.677	2.147	1.261–3.658		
Epidural anesthesia	Performed	1.157	1.054–1.270	–	–	–	–
ASA score	Class 3 or over	1.159	1.018–1.320	1.277	1.086–1.502	–	–
Colorectal cancer	Present	1.744	1.372–2.217	1.895	1.406–2.554	–	–
Metastatic or relapsed cancer	Present	1.286	1.027–1.611	–	–	1.363	1.040–1.785
Splenectomy	Performed	1.368	1.191–1.571	1.262	1.045–1.525	2.537	2.206–2.918
Pancreatectomy	Performed	2.509	2.054–3.065	–	–	8.041	6.717–9.627
Brinkman index	Over 400	1.172	1.070–1.285	1.278	1.135–1.44	1.146	1.023–1.284
Body mass index	Over 25	1.654	1.494–1.831	–	–	1.640	1.425–1.887
Body mass index category	One category–up	–	–	1.572	1.419–1.741	–	–
Serum albumin	Under 4.0 g/dl	–	–	–	–	1.177	1.057–1.309
	Under 3.5 g/dl	1.186	1.069–1.316	–	–	–	–
	Under 2.5 g/dl	–	–	1.445	1.101–1.897	–	–
Total bilirubin	Under 0.2 mg/dl	–	–	1.777	1.011–3.214	–	–
	Over 3.0 mg/dl	–	–	2.221	1.133–4.352	–	–
Aspartate aminotransferase	Over 35 IU/l	1.160	1.013–1.329	1.218	1.025–1.448	–	–
Alkaline phosphatase	Over 340 IU/l	1.213	1.053–1.399	–	–	–	–
	Over 600 IU/l	–	–	1.566	1.015–2.417	–	–
Serum Na	Under 138 mEq/l	1.135	1.001–1.286	–	–	–	–
PT/INR	Over 1.67	1.682	1.181–2.397	–	–	1.160	1.044–1.290
APTT	Under 30 s	–	–	–	–		
White blood cells	Over 9,000/μl	1.229	1.069–1.413	1.256	1.049–1.503	–	–
Body surface area	0.1 m^2^–up	–	–	–	–	1.050	1.015–1.078

Age category (<59, 60–64, 65–69, 70–74, 75–79, ≥80 years); body mass index category (<25, 25–30, 30–35, ≥35)

*ADL* activities of daily living, *APTT* activated partial thromboplastin time, *ASA* American Society of Anesthesiologists, *CI* confidence interval, *CVD* cerebrovascular disease, *OR* odds ratio, *PCI* percutaneous coronary intervention, *PT/INR* prothrombin time/international normalized ratio

**Table 4:** Risk model of nonsurgical complications

Variables	Status	Pneumonia		Prolonged ventilation		Renal failure	
		OR	95% CI	OR	95% CI	OR	95% CI
Age category 1	5 years–up	–	–	1.205	1.134–1.281	1.192	1.106–1.284
Age category 2	5 years–up	1.261	1.211–1.313	–	–	–	–
Sex	Male	2.584	2.082–3.207	1.943	1.486–2.541	–	–
Preoperative status	Emergent	–	–	1.842	1.231–2.757	–	–
Diabetes mellitus	Insulin use	1.534	1.157–2.035	–	–	–	–
Alcohol consumption	Habitual or social	–	–	–	–	1.406	1.126–1.755
Respiratory distress	Present within 30 days	1.589	1.239–2.037	1.977	1.444–2.706	–	–
Preoperative ADL	Any assistance	2.458	2.035–2.970	2.234	1.745–2.858	1.780	1.310–2.417
Preoperative ventilation	Used within 48 h	2.687	1.147–6.293	5.089	2.030–12.758	–	–
COPD	Present	1.906	1.540–2.359	1.650	1.212–2.247	2.273	1.617–3.195
Preoperative pneumonia	Performed	2.011	1.290–3.137	–	–	–	–
Congestive heart failure	Present within 30 days	–	–	–	–	1.981	1.166–3.366
Previous PCI	Performed	1.526	1.173–1.986	–	–	1.572	1.047–2.359
Previous PVD surgery	Performed	–	–	2.891	1.671–5.002	2.045	1.010–4.410
Previous CVD	Present	1.550	1.248–1.925	1.564	1.059–2.309	1.799	1.275–2.537
Acute renal failure	Present within 24 h	–	–	–	–	2.572	0.983–6.733
Steroid	Chronic use	1.999	1.289–3.100	–	–	–	–
Weight loss	More than 10%	–	–	1.443	1.128–1.846	1.533	1.138–2.066
Systemic sepsis	Present	2.091	1.194–3.662	–	–	–	–
ASA score	Class 3 or over	1.275	1.082–1.503	1.705	1.371–2.119	1.642	1.257–2.147
	Class 4 or 5	–	–	2.463	1.545–3.925	2.000	1.169–3.422
Objective of surgery	Non-cancer treatment	–	–	3.516	1.594–7.759	–	–
Esophageal cancer	Present	2.068	1.093–3.911	3.512	1.537–8.024	3.514	1.403–8.799
Gastric cancer	Present	–	–	2.141	1.047–4.379	–	–
Gallbladder cancer	Present	–	–	–	–	4.898	1.102–21.773
Splenectomy	Performed	–	–	1.576	1.172–2.119	–	–
Pancreatectomy	Performed	2.252	1.661–3.052	–	–	–	–
Brinkman index	Over 600	1.421	1.240–1.629	–	–	–	–
Body mass index	Over 25	1.256	1.039–1.518	1.869	1.455–2.399	1.401	1.062–1.847
Platelet count	Under 150,000/μl	–	–	1.352	1.041–1.756	–	–
	Under 80,000/μl	–	–	–	–	3.120	1.669–5.830
Serum albumin	Under 3.5 g/dl	1.226	1.058–1.420	–	–	1.459	1.136–1.873
	Under 3.0 g/dl	–	–	1.433	1.138–1.804	–	–
	Under 2.5 g/dl	1.531	1.179–1.989	–	–	1.504	1.021–2.214
Total bilirubin	Under 0.2 mg/dl	2.014	1.155–3.513	–	–	–	–
	Over 3.0 mg/dl	2.511	1.207–5.222	–	–	–	–
Aspartate aminotransferase	Over 35 IU/l	–	–	1.480	1.161–1.886	–	–
	Over 40 IU/l	–	––	–	–	1.815	1.327–2.482
Alkaline phosphatase	Over 600 IU/l	–	–	1.876	1.129–3.117	–	–
	Over 20 mg/dl			1.387	1.130–1.703	1.365	
Serum BUN	Over 2.0 mg/dl	–	–	–	–	1.911	
	Over 1.2 mg/dl	–	–	–	–	2.008	
eGFR	Under 30 ml/min/1.73 m^2^	–	–	–	–	1.834	1.065–3.157
Serum Na	Under 130 mEq/l	–	–	2.833	1.768–4.540	–	–
PT	Under 50%	–	–	–	–	3.233	1.653–6.322
PT/INR	Over 1.25	1.474	1.138–1.910	1.695	1.237–2.321	–	–
White blood cells	Over 9,000/μl	1.386	1.148–1.674	1.605	1.254–2.054	–	–
	Over 10,000/μl	–	–	–	–	1.818	1.238–2.671
Body surface area	0.1 m^2^–up	0.930	0.887–0.976	0.908	0.852–0.969	–	–

Age category 1 (<59, 60–64, 65–69, 70–74, 75–79, and ≥80 years); age category 2 (<59, 60–64, 65–69, 70–74, 75–79, 80–84, 85–89, and ≥90 years)

*ADL* activities of daily living, *ASA* American Society of Anesthesiologists, *BUN* blood urea nitrogen, *CI* confidence interval, *CVD* cerebrovascular disease, *eGFR* estimated glomerular filtration rate, *OR* odds ratio, *PCI* percutaneous coronary intervention, *PT* prothrombin time, *PT/INR* prothrombin time/international normalized ratio, *PVD* peripheral vascular disease

In the surgical complication risk model, sex, splenectomy, and Brinkman index were selected as common risk factors for SSI, anastomotic leak, and pancreatic fistula. Pancreatectomy was the most significant preoperative risk factor in the risk model of SSI, followed by systemic sepsis. Total bilirubin greater than 3.0 mg/dl was the most significant risk factor for anastomotic leak, followed by systemic sepsis. Similar to SSI, pancreatectomy was the most significant risk factor for pancreatic fistula, followed by splenectomy.

In the nonsurgical complication risk model, need of assistance with activities of daily living, COPD, previous CVD, ASA score class 3 and over, presence of esophageal cancer, and body mass index greater than 25 were selected as common risk factors for pneumonia, prolonged ventilation over 48 h, and renal failure. Preoperative ventilation within 48 h was the most significant risk factor in models of both pneumonia and prolonged ventilation. The presence of gallbladder cancer was the most significant risk factor for renal failure, followed by platelet count under 80,000/μl.

### Model performance

**Fig. 1:**
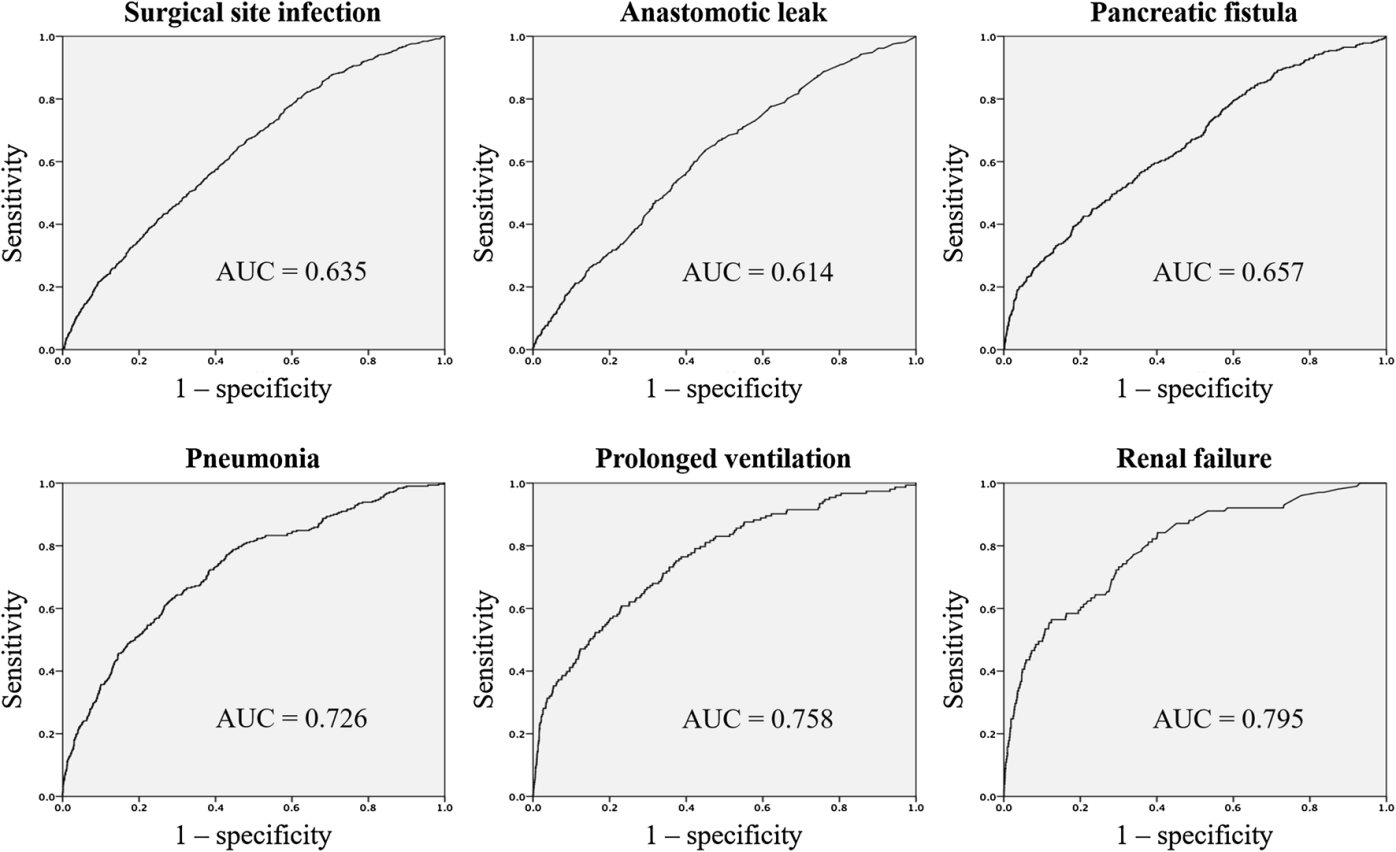
Receiver operating characteristic (ROC) curves of surgical complications (*upper panels*) and nonsurgical complications (*lower panels*) in the validation dataset consisting of 20% of the National Clinical Database (NCD) total gastrectomy population registered in 2011 and 2012. The C-index, a measure of model discrimination represented by the area under the ROC curve, was 0.635 for surgical site infection (95% CI, 0.613–0.657; *p* < 0.001), 0.614 for anastomotic leak (95% CI, 0.585–0.643; *p* < 0.001), 0.657 for pancreatic fistula (95% CI, 0.628–0.686; *p* < 0.001), 0.726 for pneumonia (95% CI, 0.697–0.755; *p* < 0.001), 0.758 for prolonged ventilation over 48 h (95% CI, 0.717–0.799; *p* < 0.001), and 0.795 for renal failure (95% CI, 0.749–0.841; *p* < 0.001)

To assess the performance of the models, both the C-index and the model calibration across risk groups were evaluated using a randomly assigned 20% of the total gastrectomy population registered between 2011 and 2012. The C-index, a measure of model discrimination represented by the area under the receiver operating characteristic (ROC) curve, was 0.635 for SSI (95% CI, 0.613–0.657; *p* < 0.001), 0.614 for anastomotic leak (95% CI, 0.585–0.643; *p* < 0.001), 0.657 for pancreatic fistula (95% CI, 0.628–0.686; *p* < 0.001), 0.726 for pneumonia (95% CI, 0.697–0.755; *p* < 0.001), 0.758 for prolonged ventilation over 48 h (95% CI, 0.717–0.799; *p* < 0.001), and 0.795 for renal failure (95% CI, 0.749–0.841; *p* < 0.001) (Fig. 1). Model calibration across risk groups was examined by comparing the observed with the predicted average within each of the five equal-sized subgroups. Calibration curves are shown in Supplementary Figure S1.

To further validate the reliability of the risk models developed and validated using the NCD data registered between 2011 and 2012, ROC curves were calculated using the NCD data registered in 2013 and the C-index was evaluated (Supplementary Figure S2). The C-index was 0.634 for SSI (95% CI, 0.618–0.649; *p* < 0.001), 0.600 for anastomotic leak (95% CI, 0.581–0.619; *p* < 0.001), 0.694 for pancreatic fistula (95% CI, 0.675–0.713; *p* < 0.001), 0.732 for pneumonia (95% CI, 0.713–0.752; *p* < 0.001), 0.750 for prolonged ventilation over 48 h (95% CI 0.720–0.779; *p* < 0.001), and 0.807 for renal failure (95% CI, 0.772–0.841; *p* < 0.001).

### Risk models for gastric cancer patients undergoing total gastrectomy

Of the NCD total gastrectomy population, most (96.7%) were gastric cancer patients; however, there were other cancer patients, and 1.6% of patients underwent total gastrectomy for diseases other than cancer. We developed preoperative risk models of six morbidities: SSI, anastomotic leak, pancreatic fistula, pneumonia, prolonged ventilation over 48 h, and renal failure for gastric cancer patients undergoing total gastrectomy. Patients without cancer, and those with esophageal cancer, colorectal cancer, gallbladder cancer, and metastatic or relapsed cancer, were excluded from the analysis.

Patient background data including age, body mass index, and preoperative activities of daily living were very similar to those of the overall total gastrectomy population (Supplementary Table S1). In the surgical complication risk model, systemic sepsis that had high ORs in the overall population was not selected as a risk factor for SSI or anastomotic leak in the gastric cancer population (Supplementary Table S2). Total bilirubin over 3.0 mg/dl or under 0.2 mg/dl was not selected as a risk factor for anastomotic leak in the gastric cancer population (Supplementary Table S2), although the former was the most significant risk factor for anastomotic leak in the overall population (Table 3). In the nonsurgical complication risk model, smoking within a year was selected as a common risk factor for pneumonia and prolonged ventilation over 48 h in the gastric cancer population (Supplementary Table S3). Chronic steroid use was a common risk factor for pneumonia, prolonged ventilation over 48 h, and renal failure with high ORs in the gastric cancer population (Supplementary Table S3). Preoperative pneumonia, systemic sepsis, or total bilirubin above 3.0 mg/dl for pneumonia, preoperative ventilation for prolonged ventilation over 48 h, and congestive heart failure or acute renal failure for renal failure were not selected as risk factors in the gastric cancer population (Supplementary Table S3).

## Discussion

Surgery has been an effective treatment for gastric cancer, and gastrectomy with D2 lymph node dissection has become a worldwide standard surgical treatment for localized gastric cancer, as several clinical trials have shown its superiority not only in Japan and South Korea but also in Europe [16–18]. However, total gastrectomy is still recognized as a difficult surgical procedure with a high morbidity and mortality rate [4]. In Japan, gastrectomy, including total gastrectomy, has been commonly performed for early and advanced gastric cancers not only in high-volume centers but also in many general hospitals with a relatively small number of gastric cancer patients. As RCTs usually enroll patients below a certain age limit without serious comorbidities in limited institutions, these studies do not entirely represent the current status of surgical procedures in Japan. In the present study, we have unveiled the current nationwide status of total gastrectomy in Japan and the first established risk models of operative morbidities for total gastrectomy.

In the present study, the C-indices of the models for the three surgical complications were lower (0.614–0.657) than those for the three nonsurgical complications (0.726–0.795). It seems that nonsurgical complications were more strongly affected by preoperative functional status, comorbidities, and laboratory data. In contrast, surgical complications can be directly affected by the surgeon’s skill and indirectly affected by operative factors such as operation time and bleeding, rather than preoperative conditions. Although other factors related to surgery such as operation time and blood loss may affect surgical complications, the present study utilized only variables that were available before the operation and could not be affected by surgeons to develop a risk model to predict surgical risk preoperatively. The NCD collaborates with the American College of Surgeons National Surgical Quality Improvement Programs (ACS-NSQIP), which shares a similar goal of developing a standardized surgery database for quality improvement [19]. Patient variables and definitions were almost identical to those used by the ACS-NSQIP.

In a previous study, the nationwide mortality rates after total gastrectomy were first reported to be satisfactory using the NCD data in 2011, and a risk model of mortality was established for Japanese patients undergoing total gastrectomy [13]. The mortality and morbidity rates in the current study utilizing the NCD data in 2011 and 2012 were comparable with those in the previous study utilizing the 2011 data alone, suggesting that the characteristics of total gastrectomy in Japan as well as the NCD data collection are stable and reliable. In addition, it should be noted that Japanese surgeons are very familiar with gastrectomy because gastric cancer is one of the most commonly encountered diseases in Japanese surgical units. These facts potentially explain why such a large amount of patient data from a large number of institutes was constantly corrected and was of high quality throughout the 2 years.

Among surgical complications after total gastrectomy, SSI was the most frequent, followed by pancreatic fistula and anastomotic leak. Pneumonia and transfusion were the most frequent nonsurgical complications after total gastrectomy, followed by systemic sepsis and prolonged ventilation over 48 h. Pancreatectomy was the most significant factor in both risk models of SSI and pancreatic fistula. Because surgical complication is strongly influenced by the surgical procedure itself, it is understandable that surgical procedures such as pancreatectomy and splenectomy were selected as risk factors. During the total gastrectomy procedure, splenectomy was usually added for D2 lymph node dissection in Japan, including the splenic hilar lymph node, although European RCTs comparing D1 and D2 lymphadenectomy for gastric cancer demonstrated significantly increased morbidity and mortality with splenectomy or pancreatosplenectomy in D2 total gastrectomy [20, 21]. In contrast, pancreatectomy is sometimes inevitable because of tumor invasion into the pancreas or may be performed as combined pancreatosplenectomy for extended lymph node dissection in some cases. In the NCD population, splenectomy and pancreatectomy were performed for 7.8% and 2.1% of patients undergoing total gastrectomy, respectively. In contrast, previous reports from Western countries have reported higher rates of splenectomy and pancreatectomy that were performed simultaneously with gastrectomy [12, 22]. Although it is difficult to compare clinical data in other countries with the NCD data, it seems that a relatively smaller number of patients undergo splenectomy with or without pancreatectomy simultaneously with gastrectomy in Japan, which is perhaps related to differences in patient characteristics, including tumor stage. The number of patients undergoing splenectomy simultaneously with gastrectomy may be further decreasing in the future. In a multi-institutional RCT in Japan, the noninferiority of spleen preservation to splenectomy, regarding overall survival, has been recently confirmed for patients with proximal gastric cancer not invading the greater curvature [23].

Of the NCD overall total gastrectomy population, the great majority (96.7%) consisted of gastric cancer patients. Additionally, a small portion of patients had esophageal cancer (0.5%), colorectal cancer (1.8%), or gallbladder cancer (0.1%) at the time of total gastrectomy, and had simultaneously undergone operations for those cancers. In addition, those cancers were selected as risk factors for operative morbidities: colorectal cancer for SSI and anastomotic leak, esophageal cancer for the three nonsurgical complications, and gallbladder cancer for renal failure. Although the occurrence rates were relatively low, those synchronous cancers appear to be significant risk factors for surgical and nonsurgical complications with high ORs. In the risk models for gastric cancer patients undergoing total gastrectomy, those factors were not selected naturally because patients with other cancers were excluded from the analysis.

Japan is known for its longevity, with an increasing percentage of the population aged >80 years: 7.1% in 2012 compared with 0.7% in 1960 [24]. Inevitably, an increasing number of elderly patients have undergone gastrectomy, including total gastrectomy, in recent years [8]. It has been reported that elderly patients are more likely to have comorbidities compared with young patients [25, 26]. Consistent with previous reports, age category was the risk factor for five morbidities, other than pancreatic fistula, of six morbidities analyzed in the present study [12, 25–27]. On the other hand, it is likely that less-invasive surgery would be selected for elderly patients with minimal lymph node dissection compared with younger patients. Therefore, further studies are needed to clarify the relationship between age and operative risk.

In our earlier report, the most important variable affecting 30-day and overall operative mortality rates after total gastrectomy was the ASA score [13]. In contrast, the ASA score minimally affected the risk model of either surgical complications or nonsurgical complications in the current study. It has been suggested that the ASA score is more likely to represent the patient’s general condition, which can affect operative tolerability or survival, based on its advantages of simplicity and universal use as an effective risk indicator.

There are some limitations in the use of the NCD dataset, including a lack of clinical information such as tumor location, intraoperative factors such as bleeding, and the extent of lymphadenectomy. In addition, our study does not capture known long-term complications or postoperative sequelae such as dumping syndrome or malnutrition, because data collection was terminated 30 days after the operation in each case. However, our study highlights the current status of total gastrectomy in Japan, and the created risk models of operative morbidities are expected to be useful tools to preoperatively predict short-term outcomes of total gastrectomy and to compare the quality of surgical outcomes in a risk-adjusted fashion.

In conclusion, we have created the first reported risk models of morbidities associated with total gastrectomy, using a Japanese nationwide database. The total gastrectomy mortality and morbidity rates in the nationwide population-based cohort were satisfactory. The risk models developed in this study may be useful to preoperatively predict operative morbidities and to compare the quality of surgical outcomes in a risk-adjusted fashion in patients undergoing total gastrectomy.

## Electronic supplementary material

Below is the link to the electronic supplementary material.

Supplementary material 1 (PDF 451 kb)

Supplementary material 2 (DOCX 47 kb)
