# Supplementary material for: Development and external validation of preoperative risk models for operative morbidities after total gastrectomy using a Japanese web-based nationwide registry
Source: Gastric Cancer. 2017 Mar 11;20(6):987–97. doi: 10.1007/s10120-017-0706-9 (PMC5658454; doi:10.1007/s10120-017-0706-9)
Supplement: Supplementary file 1 — Supplementary material 1 (PDF 451 kb) [file 10120_2017_706_MOESM1_ESM.pdf]

Supplementary Figures

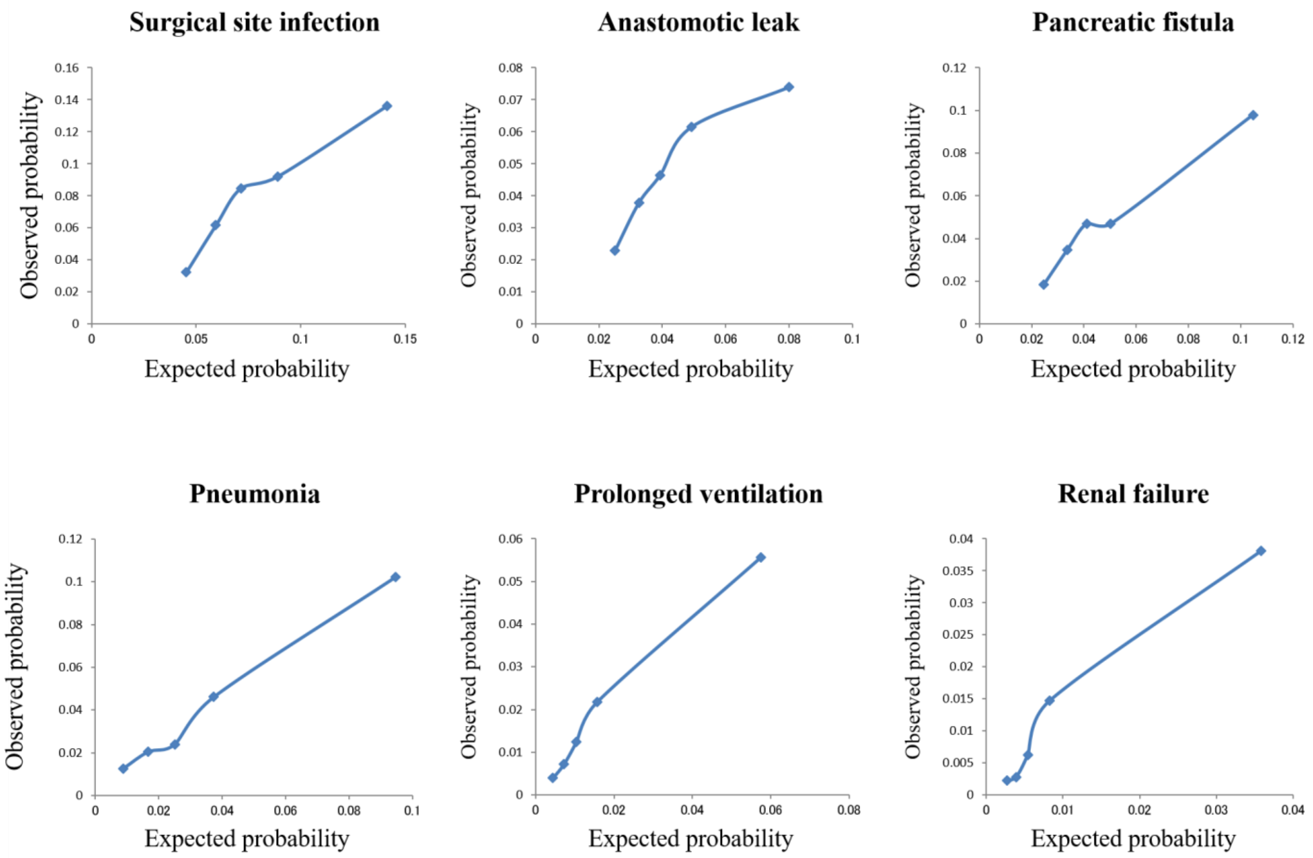

Supplementary Figure S1. Calibration curves showing the observed and expected morbidity rates for each quintile for surgical complications (upper panels) and non-surgical complications (lower panels) in the NCD total gastrectomy population. Each risk quintile contains approximately 1,500 patients. Model calibration, defined as the degree to which the observed outcomes were similar to the predicted outcomes, was examined by comparing the observed with the predicted average within each of the five equal-sized subgroups, arranged in increasing the order of patient risk.

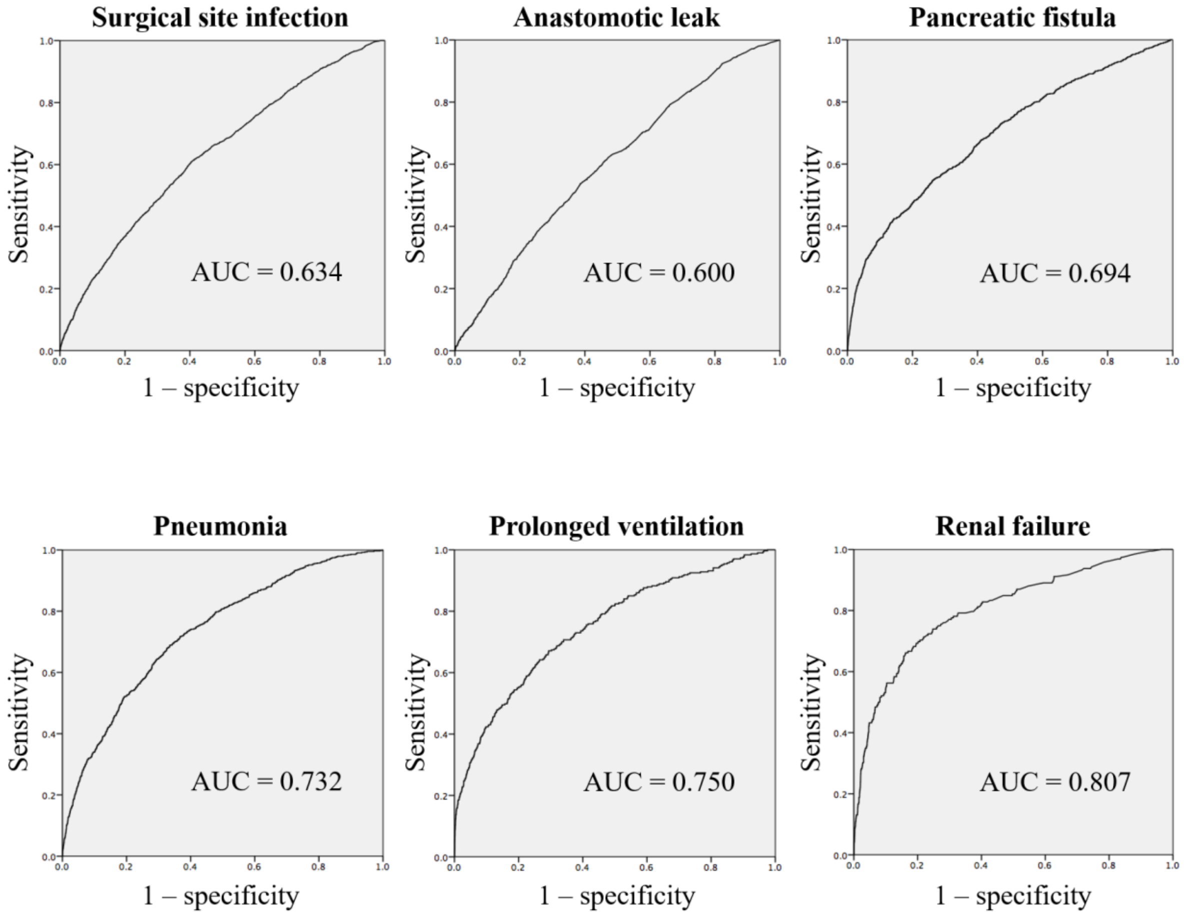

Supplementary Figure S2. Receiver operating characteristic (ROC) curves of surgical complications (upper panels) and non-surgical complications (lower panels) in the NCD total gastrectomy population registered in 2013. The C-index, a measure of model discrimination represented by the area under the ROC curve, was 0.634 for surgical site infection (95% CI, 0.618–0.649; $p < 0.001$), 0.600 for anastomotic leak (95% CI, 0.581–0.619; $p < 0.001$), 0.694 for pancreatic fistula (95% CI, 0.675–0.713; $p < 0.001$), 0.732 for pneumonia (95% CI, 0.713–0.752; $p < 0.001$), 0.750 for prolonged ventilation over 48 hours (95% CI, 0.720–0.779; $p < 0.001$), and 0.807 for renal failure (95% CI, 0.772–0.841; $p < 0.001$).
