# Supplementary material for: Development and external validation of preoperative risk models for operative morbidities after total gastrectomy using a Japanese web-based nationwide registry
Source: Gastric Cancer. 2017 Mar 11;20(6):987–97. doi: 10.1007/s10120-017-0706-9 (PMC5658454; doi:10.1007/s10120-017-0706-9)
Supplement: Supplementary file 2 — Supplementary material 2 (DOCX 47 kb) [file 10120_2017_706_MOESM2_ESM.docx]

**Supplementary Table S1**: Key descriptive data of patients undergoing total gastrectomy for gastric cancer.

| **Variables** | **N = 36,017** |
| --- | --- |
| Age, mean ± SD, years  Age category, total  <59 years (%)  60–64 years (%)  65–69 years (%)  70–74 years (%)  75–79 years (%)  ≥80 years (%)  Sex, male (%)  Body surface area, mean ± SD, m^2^  Body mass index, category, total  <25 (%)  25–30 (%)  30–35 (%)  ≥35 (%)  Emergent surgery (%)  Diabetes mellitus (%)  Smoking within a year (%)  Habitual alcohol consumption (%)  Any alcohol consumption (%)  Respiratory distress within 30 days (%)  Preoperative ADL, any assistance (%)  Preoperative ventilation within 48 hours (%)  COPD (%)  Preoperative pneumonia (%)  Ascites within 30 days (%)  Ascites, uncontrolled (%)  Hypertension within 30 days (%)  Hypertension without treatment (%)  Congestive heart failure within 30 days (%)  Myocardial infarction within 6 months (%)  Angina pectoris within 30 days (%)  Previous PCI (%)  Previous cardiac surgery (%)  Previous PVD surgery (%)  Previous cerebrovascular disease (%)  Previous cerebrovascular accident (%)  Acute renal failure within 24 hours (%)  Preoperative dialysis within 14 days (%)  Disseminated cancer (%)  Chronic steroid use (%)  Weight loss over 10% within 6 months (%)  Bleeding disorder (%)  Preoperative transfusion within 72 hours (%)  Preoperative chemotherapy within 30 days (%)  Systemic sepsis (%)  Epidural anesthesia (%)  ASA score, class 3 and over (%)  ASA score, class 4 and 5 (%)  Cholecystectomy (%)  Splenectomy (%)  Pancreatectomy (%) | 69.1 ± 11.0  36,017  5,749 (16.0)  5,285 (14.7)  5,712 (15.9)  7,011 (19.5)  6,595 (18.3)  5,665 (15.7)  26,620 (73.9)  1.58 ± 0.19  34,927  29,129 (83.4)  5,213 (14.9)  494 (1.4)  91 (0.3)  450 (1.2)  5,727 (15.9)  7,685 (21.3)  9,283 (25.8)  18,916 (52.5)  809 (2.2)  1,586 (4.4)  23 (0.1)  1,514 (4.2)  151 (0.4)  660 (1.8)  555 (1.5)  12,088 (33.6)  789 (2.2)  253 (0.7)  263 (0.7)  561 (1.6)  981 (2.7)  412 (1.1)  215 (0.6)  1,392 (3.9)  717 (2.0)  20 (0.1)  199 (0.6)  1,005 (2.8)  279 (0.8)  2,970 (8.2)  1,240 (3.3)  1,345 (3.7)  1,421 (3.9)  90 (0.2)  25,101 (69.7)  3,493 (9.7)  167 (0.5)  6,633 (18.4)  2,793 (7.8)  576 (1.6) |

ADL, activities of daily living; ASA, American Society of Anesthesiologists; COPD, chronic obstructive pulmonary disease; PCI, percutaneous coronary intervention; PVD, peripheral vascular disease; SD, standard deviation

**Supplementary Table S2**: Risk model of surgical complications in the gastric cancer population

| **Variables** | **Status** | **Surgical site infection** | | **Anastomotic leak** | | **Pancreatic fistula** | |
| --- | --- | --- | --- | --- | --- | --- | --- |
|  |  | **OR** | **95% CI** | **OR** | **95% CI** | **OR** | **95% CI** |
| Age category  Sex  Alcohol consumption  Preoperative ADL  Ascites  Hypertension  Previous PCI  Previous CVD  Steroid  Weight loss  Epidural anesthesia  ASA score  Splenectomy  Pancreatectomy  Brinkman index  Body mass index  Platelet count  Serum albumin  Aspartate aminotransferase  Alkaline phosphatase  Serum Na  PT  APTT  White blood cells  Body surface area | 5 years-up  male  habitual or social  any assistance  present  present within 30 days  performed  present  chronic use  over 10%  performed  class 5 or over  class 3 or over  performed  performed  over 400  over 25  over 30  under 80,000/μl  under 4.0 g/dl  under 3.5 g/dl  under 2.5 g/dl  over 35 IU/l  over 340 IU/l  under 138 mEq/l  under 50%  under 30 sec  over 12,000 /μl  over 9,000 /μl  0.1 m^2^-up | –  1.373  1.102  1.372  –  1.220  –  1.392  1.958  1.321  1.172  –  1.185  1.349  2.422  1.217  1.592  –  1.698  –  1.194  –  1.155  1.224  1.148  1.702  –  1.376  –  – | –  1.231 – 1.531  1.012 – 1.200  1.159 – 1.624  –  1.125 – 1.324  –  1.174 – 1.651  1.402 – 2.734  1.162 – 1.501  1.074 – 1.280  –  1.048 – 1.340  1.184 – 1.537  1.940 – 3.023  1.117 – 1.326  1.446 – 1.752  –  1.142 – 2.525  –  1.082 – 1.318  –  1.015 – 1.314  1.068 – 1.403  1.018 – 1.296  1.113 – 2.602  –  1.078 – 1.757  –  – | 1.063  1.340  –  1.411  1.422  1.188  1.350  –  2.155  1.203  –  2.788  1.197  1.222  –  1.234  1.714  1.592  –  –  –  1.336  1.246  –  –  –  –  –  1.270  – | 1.028 – 1.098  1.169 – 1.536  –  1.144 – 1.741  1.038 – 1.949  1.066 – 1.324  1.050 – 1.737  –  1.443 – 3.219  1.011 – 1.431  –  1.270 – 6.119  1.021 – 1.403  1.021 – 1.463  –  1.103 – 1.380  1.510 – 1.945  1.184 – 2.139  –  –  –  1.006 – 1.772  1.059 – 1.466  –  –  –  –  –  1.064 – 1.515  – | –  1.358  1.253  –  –  –  –  –  1.826  –  –  –  –  2.655  7.355  1.158  1.558  –  –  1.127  –  –  –  –  –  –  1.217  –  –  1.049 | –  1.163 – 1.585  1.125 – 1.396  –  –  –  –  –  1.172 – 2.843  –  –  –  –  2.329 – 3.026  6.040 – 8.957  1.041 - 1.288  1.366 – 1.778  –  –  1.019 – 1.247  –  –  –  –  –  –  1.102 – 1.344  –  –  1.016 – 1.082 |

Age category (<59, 60–64, 65–69, 70–74, 75–79, and ≥80 years).

ADL, activities of daily living; APTT, activated partial thromboplastin time; ASA, American Society of Anesthesiologists; CI, confidence interval; CVD, cerebrovascular disease; OR, odds ratio; PCI, percutaneous coronary intervention; PT, prothrombin time.

**Supplementary Table S3**: Risk model of non-surgical complications in the gastric cancer population

| **Variables** | **Status** | **Pneumonia** | | **Prolonged ventilation** | | **Renal failure** | |
| --- | --- | --- | --- | --- | --- | --- | --- |
|  |  | **OR** | **95% CI** | **OR** | **95% CI** | **OR** | **95% CI** |
| Age category 1  Age category 2  Sex  Preoperative status  Diabetes mellitus  Alcohol consumption  Smoking  Respiratory distress  Preoperative ADL  Preoperative ventilation  COPD  Ascites  Hypertension  Myocardial infarction  Previous PCI  Previous PVD surgery  Previous CVD  Disseminated cancer  Steroid  Weight loss  Bleeding disorder  ASA score  Splenectomy  Pancreatectomy  Brinkman index  Body mass index  Platelet count  Serum albumin  Total bilirubin  Aspartate aminotransferase  Alkaline phosphatase  Serum BUN  Serum creatinine  eGFR  Serum Na  PT  PT/INR  White blood cells  Body surface area | 5 years-up  5 years-up  male  emergent  insulin use  habitual or social  within a year  present within 30 days  any assistance  used within 48 hours  present  present  present within 30 days  present within 6 months  performed  performed  present  present  chronic use  over 10%  present  class 3 or over  performed  performed  over 600  over 25  under 120,000/μl  under 80,000/μl  under 3.5 g/dl  under 3.0 g/dl  under 2.5 g/dl  under 0.2 mg/dl  over 3.0 mg/dl  over 35 IU/l  over 40 IU/l  over 340 IU/l  over 20 mg/dl  over 1.2 mg/dl  over 2.0 mg/dl  under 30 ml/min/1.73m^2^  under 130 mEq/l  under 50%  over 1.25  over 9,000/μl  over 12,000/μl  0.1 m^2^-up | –  1.284  2.130  –  1.331  –  1.242  1.539  2.265  5.056  2.132  –  1.191  –  1.306  1.873  1.553  –  2.475  1.232  –  1.248  –  2.118  1.209  –  1.493  –  1.292  –  1.593  1.949  –  –  –  1.209  –  –  –  –  –  –  1.559  1.287  –  – | –  1.237 - 1.333  1.785- 2.541  –  1.003 – 1.765  –  1.068 – 1.443  1.214 – 1.951  1.888 – 2.717  1.906 – 13.410  1.758 – 2.585  –  1.057 – 1.341  –  1.010 – 1.690  1.216 – 2.885  1.264 – 1.907  –  1.655 – 3.701  1.033 – 1.468  –  1.068 – 1.459  –  1.504 – 2.982  1.049 – 1.393  –  1.144 – 1.948  –  1.126 – 1.481  –  1.237 – 2.052  1.115 – 3.407  –  –  –  1.001 – 1.459  –  –  –  –  –  –  1.222 – 1.989  1.067 – 1.552  –  – | 1.257  –  1.759  1.705  –  –  1.296  2.018  1.900  –  1.616  –  –  1.794  –  2.642  1.522  –  1.829  1.585  –  1.718  1.493  –  –  1.776  1.556  –  –  1.646  –  –  –  1.594  –  –  –  1.430  –  –  4.208  –  1.938  –  2.082  0.927 | 1.183 – 1.336  –  1.350 – 2.293  1.115 – 2.609  –  –  1.062 – 1.581  1.490 – 2.733  1.484 – 2.433  –  1.216 – 2.147  –  –  1.067 – 3.015  –  1.567 – 4.452  1.049 – 2.209  –  1.015 – 3.297  1.261 – 1.994  –  1.400 – 2.109  1.125 – 1.980  –  –  1.402 – 2.250  1.103 – 2.196  –  –  1.321 – 2.051  –  –  –  1.268 – 2.003  –  –  –  1.123 – 1.820  –  –  2.779 – 6.372  –  1.435 – 2.616  –  1.436 – 3.020  0.871 – 0.986 | 1.209  –  –  –  –  1.287  –  1.795  1.552  –  1.963  1.636  1.289  –  1.669  1.966  1.646  1.729  2.335  1.498  2.005  1.629  1.441  –  1.274  1.663  –  3.085  –  1.758  –  –  2.814  –  1.831  –  1.358  –  2.190  3.456  2.409  2.626  –  –  2.026  – | 1.123 – 1.301  –  –  –  –  1.039 – 1.594  –  1.234 – 2.613  1.137 – 2.118  –  1.390 – 2.774  1.024 – 2.614  1.038 – 1.601  –  1.141 – 2.440  0.998 – 3.872  1.172 – 2.311  1.155 – 2.588  1.226 – 4.446  1.121 – 2.003  1.042 – 3.857  1.270 – 2.090  1.000 – 2.077  –  1.012 – 1.603  1.290 – 2.143  –  1.607 – 5.924  –  1.346 – 2.296  –  –  0.995 – 7.957  –  1.350 – 2.482  –  1.039 – 1.775  –  1.434 – 3.344  2.427 – 4.921  1.280 – 4.534  1.278 – 5.396  –  –  1.282 – 3.203  – |

Age category 1 (<59, 60–64, 65–69, 70–74, 75–79, and ≥80 years); Age category 2 (<59, 60–64, 65–69, 70–74, 75–79, 80–84, 85–89, and ≥90 years).

ADL, activities of daily living; ASA, American Society of Anesthesiologists; BUN, blood urea nitrogen; CI, confidence interval; CVD, cerebrovascular disease; eGFR, estimated glomerular filtration rate; OR, odds ratio; PCI, percutaneous coronary intervention; PT, prothrombin time; PT/INR, prothrombin time/international normalized ratio; PVD, peripheral vascular disease.
